# The Tumor Suppressor Roles of MYBBP1A, a Major Contributor to Metabolism Plasticity and Stemness

**DOI:** 10.3390/cancers12010254

**Published:** 2020-01-20

**Authors:** Blanca Felipe-Abrio, Amancio Carnero

**Affiliations:** 1 Instituto de Biomedicina de Sevilla, IBIS, Hospital Universitario Virgen del Rocío, Universidad de Sevilla, Consejo Superior de Investigaciones Científicas, Avda. Manuel Siurot s/n, 41013 Seville, Spain; blanca.felipeabrio@kuleuven.vib.be; 2 CIBERONC, Instituto de Salud Carlos III, 28029 Madrid, Spain

**Keywords:** tumor suppressor, MYBBP1A, MYB, metabolic plasticity, stemness, OXPHOS, PGC1-α, VHL, renal cancer

## Abstract

The MYB binding protein 1A (MYBBP1A, also known as p160) acts as a co-repressor of multiple transcription factors involved in many physiological processes. Therefore, MYBBP1A acts as a tumor suppressor in multiple aspects related to cell physiology, most of them very relevant for tumorigenesis. We explored the different roles of MYBBP1A in different aspects of cancer, such as mitosis, cellular senescence, epigenetic regulation, cell cycle, metabolism plasticity and stemness. We especially reviewed the relationships between MYBBP1A, the inhibitory role it plays by binding and inactivating c-MYB and its regulation of PGC-1α, leading to an increase in the stemness and the tumor stem cell population. In addition, MYBBP1A causes the activation of PGC-1α directly and indirectly through c-MYB, inducing the metabolic change from glycolysis to oxidative phosphorylation (OXPHOS). Therefore, the combination of these two effects caused by the decreased expression of MYBBP1A provides a selective advantage to tumor cells. Interestingly, this only occurs in cells lacking pVHL. Finally, the loss of MYBBP1A occurs in 8%–9% of renal tumors. tumors, and this subpopulation could be studied as a possible target of therapies using inhibitors of mitochondrial respiration.

## 1. Introduction

MYBBP1A is a ubiquitous 160 kDa protein, first identified for its binding and repression of the proto-oncogene c-MYB. Since then, it has been found that MYBBP1A acts as a co-repressor of multiple transcription factors involved in many important physiological processes. In all these processes, MYBBP1A acts as a tumor suppressor, regulating the evolution and malignity of cells. Furthermore, the loss of MYBBP1A occurs in 8%–9% of renal tumors and may occur in a percentage of other tumors, according to its role as a potent tumor suppressor. However, to date, there has been no systematic review of the roles of MYBBP1A, and how it may control these processes. And more importantly, there is no thorough review of how the loss of MYBBP1A contributes to the development and evolution of tumors. It also will be interesting to explore whether this subpopulation could be studied as a possible target of new therapies. In this review, we will review these points with a special interest in the relationship between stemness and metabolic plasticity.

## 2. Tumor Suppressor Roles of MYBBP1A 

### 2.1. MYB Binding Protein 1A

**Figure 1:**
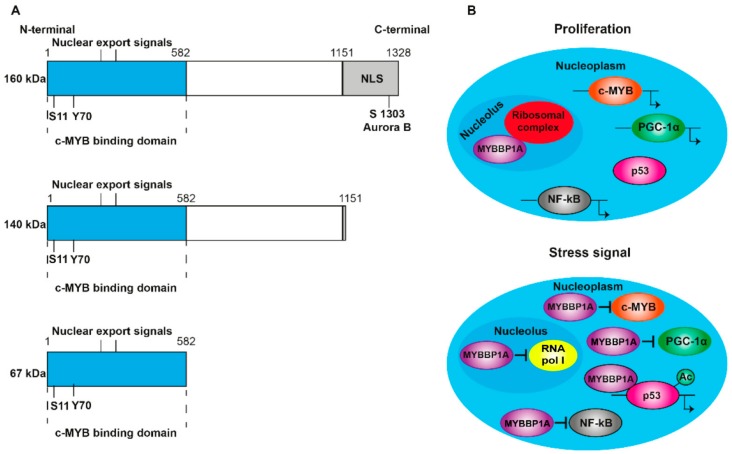
Structure and translocation model of MYBBP1A. (**A**) Scheme of the domains and phosphorylation sites of MYBBP1A and their shortened forms, produced by proteolytic cleavage. NLS: nuclear and nucleolar localization sequence. S1303 Aurora B indicates that MYBBP1A is phosphorylated by Aurora B kinase at serine residue 1303. ARNpolI is RNA polymerase I. (**B**) Model, proposed by Hochstatter et al., suggests that MYBBP1A is located in the nucleolus of the proliferative cells attached to the ribosomal complex. However, in cells subjected to some stress signals, such as glucose deprivation, MYBBP1A translocates mainly to the nucleoplasm, where it binds to different transcription factors to interrupt cell cycle progression, proliferation and energy production.

The MYB binding protein 1A (MYBBP1A, also known as p160) is a 160 kDa protein that is expressed in all tissues. It is located predominantly in the nucleolus, although its presence in the nucleoplasm has also been observed [1]. It contains nuclear and nucleolar localization sequences (NLS) at the C-terminal end (Figure 1A) and is anchored to the nucleolus through its interaction with RNA. MYBBP1A translocates from the nucleolus to the nucleoplasm when there is a decrease in the RNA content caused by stress signals, such as the absence of glucose or the inhibition of ribosome synthesis [2,3]. In addition, ribosomal stress induces the processing of MYBBP1A in its shortened forms (140 and 67 kDa) (Figure 1A), which are found in the nucleoplasm following the loss of the nucleolar localization signal. Although p160 is expressed in al tissues, the 67 kDa protein is only expressed in a few subcultivated cell lines. MYBBP1A is a highly evolutionarily conserved protein, presenting some homology with the Pol5p protein of Saccharomyces cerevisiae, and is involved in the production of ribosomal RNA (rRNA) [4]. Initially, it was identified as a protein that was bound to the protein encoded by the proto-oncogene c-MYB [1], and, subsequently, its binding to numerous transcription factors has been discovered. For this reason, it is thought that the function of MYBBP1A is the regulation of the activity of several transcription factors, acting as a regulator of fundamental biological processes such as cell division, proliferation and apoptosis.

Mori et al. [5] deleted the *mybbp1a* gene in mice and obtained *mybbp1a* +/- heterozygous healthy and fertile mice but failed to generate any *mybbp1a* -/- mice. After analyzing the embryos in depth at different points of development (days 6.5, 9.5 and 11.5), they found no homozygous *mybbp1a* -/- embryos. They also isolated blastocysts from heterozygous mice, kept them in culture for several days and genotyped them without detecting any *mybbp1a* -/- blastocysts. Therefore, although the molecular mechanism involved is unclear, *mybbp1a* is essential in early embryonic development. 

### 2.2. Regulation of MYBBP1A

MYBBP1A is a ubiquitination target of the von Hippel–Lindau tumor suppressor gene (VHL) [6], which is a component of the E3 ubiquitin ligase complex. The loss of function mutations in this gene is associated with von Hippel–Lindau disease, a hereditary cancer syndrome characterized by a high risk of developing highly vascularized tumors in the eyes, brain and spine, as well as benign or malignant tumors in the kidney, pancreas and adrenal medulla [7]. The biallelic inactivation of VHL is also frequent in renal carcinomas and sporadic hemangioblastomas [7,8]. It has been found that the VHL protein (pVHL) binds directly to MYBBP1A, causing its degradation in an iron- and proteasome-dependent manner [6]. The region of MYBBP1A, located between amino acids 632 and 694, is necessary for this degradation to occur. This region contains a sequence similar to the one surrounding the hydroxylated proline in the HIF1α protein that is required for its degradation, which also occurs via pVHL. It has also been found that the mutation in the proline residue 693 of MYBBP1A (Pro693), when changed to alanine, blocks the degradation of MYBBP1A by pVHL. Moreover, as in HIF1α degradation, MYBBP1A degradation by pVHL also requires iron, as iron chelation with desferrioxamine (DFO) abolishes it. Based on these findings, it has been proposed that the Pro693 residue of MYBBP1A is hydroxylated in the presence of iron and oxygen, leading to its ubiquitination by pVHL and its subsequent degradation via the proteasome. Despite the similarities between MYBBP1A and HIF1α degradation by pVHL in terms of amino acid sequence and iron requirement, how hypoxia affects MYBBP1A’s stability at protein and mRNA levels is still unknown.

**Figure 2:**
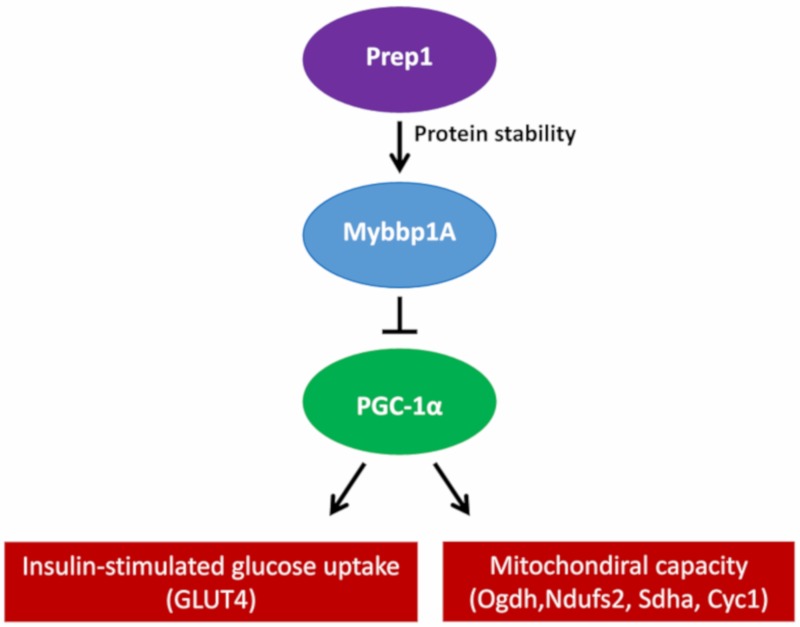
Proposed mechanism of how Prep1 regulates Mybbp1a stability, modulating Pgc-1α, mitochondrial genes and Glut 4 expression in mouse skeletal muscle.

However, pVHL is not the only protein involved in the regulation of MYBBP1A. PREP1 controls the half-life of MYBBP1A at the protein stability level, since the interaction between PREP1 and MYBBP1A prevents its degradation via the proteasome. The muscles of hypomorphic mice for Prep1 show reduced levels of Mybbp1A, but an increase in the peroxisome proliferator-activated receptor γ coactivator (Pgc-1α), a well-known regulator of mitochondrial metabolism and biogenesis. Pgc-1α upregulation occurs at both the mRNA and protein levels, accompanied by an increase in the expression of the Glut4 transporter. Conversely, this effect is revoked by the direct delivery of Mybbp1A cDNA in the muscle of Prep1-deficient mice, decreasing Pgc-1α and Glut4 expression [9]. Furthermore, the elimination of Prep1 specifically in the muscles of mice causes an increase in the expression of the genes of the respiratory chain and Pgc-1α, together with a significant reduction in the levels of Mybbp1a (Figure 2) [10]. 

**Figure 3:**
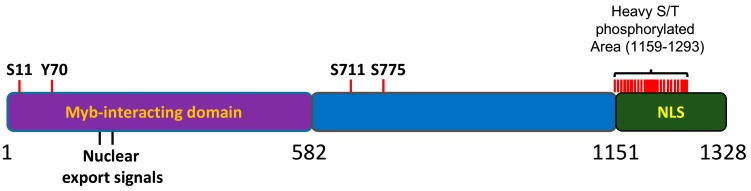
Schematic representation of MYBBP1A protein. 1-582aa domain, reported to interact with the Myb protein. NLS: nuclear and nucleolar localization signal. S, T or Y are the phophorylation residues reported in several phosphoproteomic assays. Adapted from the atlas of genetics and cytogenetics in oncology and haematology.

MYBBP1A has been found to be posttranslationally modified; however, the biological relevance of these modifications has not yet been clarified. Importantly, the MYBBP1A protein is heavily phosphorylated in cells [11,12,13,14]. Most of the sites for phosphorylation (18 out of 21) are mapped to the C terminal region of the protein, within a 200 aa portion that has been found to be relevant for the nucleolar localization of the protein [15] (Figure 3). Accordingly, MYBBP1A was found to be an important component of the phosphoproteome of the mitotic spindle; however, the Aurora B kinase is the only proven kinase acting on the protein in vitro [16]. MYBBP1A can be phosphorylated by different kinases, but scarce data have been shown for many of them.

### 2.3. MYBBP1A as a Regulator of Transcription Factors

MYBBP1A was first identified because of its binding to the proto-oncogene c-MYB [1]. The proto-oncogene c-MYB codes for the transcription factor c-MYB, which is an essential regulator of stem cells and progenitors of the bone marrow, colon crypts and neurogenic regions of the adult brain [17]. Its main function is to maintain the proliferative state and the immature characteristics of these cells. The oncogenic forms of this protein are generated by loss of the N-terminus, C-terminus or both, maintaining the DNA binding domain and the region necessary for the transactivation of transcription. At the C-terminal end is the negative regulatory domain (NRD), which negatively regulates transactivation and the binding of the protein to DNA [18,19]. Both MYBBP1A and its shortened 67 kDa form, which are generated by proteolytic cleavage in some cell types, bind to the NRD of c-MYB. This binding occurs through the leucine zipper motifs present in the N-terminal fragment of MYBBP1A and in the NRD of c-MYB [1].

It has also been found that MYBBP1A acts as a repressor of PGC-1α. Both the N-terminal and the C-terminal domain of MYBBP1A bind to the negative regulatory domain of PGC-1α and reduce its ability to stimulate mitochondrial respiration and the expression of electron transport chain genes. This negative regulation is inhibited when PGC-1α is phosphorylated by the p38 MAP kinase [20]. Another transcription factor regulated by MYBBP1A is PREP1 [21]. Both the 160 kDa and the 67 kDa version of MYBBP1A bind to the first homology domain (HR1) of PREP1, inhibiting its transcriptional activity and the expression of the *HoxB2* gene in NT2-D1 cells treated with retinoic acid. MYBBP1A competes with PBX1 to bind to PREP1 [21]. MYBBP1A also competes with p300 for binding to the RelA/p65 subunit of NF-κB, acting as a repressor of its activity when the binding occurs [22]. In addition, MYBBP1A acts as a corepressor of CRY1 in the period2 promoter, inhibiting the expression of this gene, which is involved in the regulation of the circadian clock [23]. Despite its general role as a negative regulator of transcription factors, MYBBP1A also acts as an activator of some transcription factors, such as the aromatic hydrocarbon receptor (AhR) [24].

### 2.4. MYBBP1A and Epigenetic Regulation

MYBBP1A also binds to factors involved in epigenetic regulation. It has been found that MYBBP1A is a component of the ATP-dependent chromatin remodeling complex (esBAF), which is associated with differentiation processes and stem cell phenotypes. Although the role of MYBBP1A in this complex is not clear, it could be related to epigenetic regulatory functions [25,26]. Likewise, it has been shown that MYBBP1A acts as a regulator of muscle-specific gene expression and the differentiation of myoblasts by binding to MyoD. The binding of MYBBP1A and MyoD in certain regions of genes that are not expressed in muscle tissue decreases the transcriptional activity of MyoD. It has been proposed that MYBBP1A carries out this repressive function by recruiting negative epigenetic modifiers, such as Histone Deacetylase 1 and 2, HDAC1/2 and Suv39h1, inducing a less accessible structure of chromatin [27].

It has also been found that MYBBP1A plays an important role in the acetylation and activation of p53 [2,28,29,30,31,32]. Under stress conditions in the nucleolus, which are produced by the inhibition of the ribosome synthesis or by the absence of glucose, rRNA transcription is blocked, and the RNA content in the nucleolus is reduced. This decrease causes the translocation of MYBBP1A to the nucleoplasm, where it facilitates the interaction between p53 and p300, increasing the acetylation of p53. Specifically, the proposed model states that stress in the nucleolus causes the inactivation of Human Double Minute 2, HDM2, which leads to the stabilization of p53 and the formation of non-acetylated p53 dimers. Once translocated to the nucleoplasm, MYBBP1A binds to these p53 dimers through its central region (aa 643–1150) and C-terminal end (aa 1151–1328). After this interaction occurs, dimers are formed between units of MYBBP1A bound to p53 dimers, resulting in the formation of p53 tetramers. The transcription coactivator p300 binds to these structures, acetylates the p53 units, and then MYBBP1A dissociates from the complex. Both MYBBP1A-p53 binding sites (aa 643–1150 and aa 1151–1328) are required to facilitate p53 tetramerization and acetylation, so that only full-length MYBBP1A is able to promote p53 activation. Finally, the tetramer of acetylated p53, still bound to p300, binds to the promoters of its target genes and induces the expression of genes related to apoptosis and/or cell cycle arrest [31].

### 2.5. Role of MYBBP1A in Cell Cycle and Mitosis

MYBBP1A has been linked to cell cycle control, especially the G2/M phase, and mitosis. On the one hand, MYBBP1A silencing in HeLa cells induces the up-regulation of genes that inhibit growth, such as BRCA1, TOP2A and WEE1, and the down-regulation of genes involved in DNA repair, such as CDKN1a and GADD45b, ultimately provoking a cell cycle arrest at G2/M [5]. On the other hand, there are several findings suggesting that MYBBP1A participates in the regulation of mitosis. It has been found that MYBBP1A translocates from the nucleolus to the nucleoplasm during mitosis, situating itself in parachromosomal regions in metaphase–anaphase, where it colocalizes with the chromatin marker phosphohistone H3 [5]. In addition, MYBBP1A is phosphorylated by Aurora B kinase at serine residue 1303. The peak of this phosphorylation occurs at the G2/M transition and is completely inhibited when cells are treated with danusertib, an inhibitor of Aurora kinases. This phosphorylation of MYBBP1A in G2/M is performed exclusively by the Aurora B kinase, since only the silencing of Aurora B (and not A or C) suppresses this phosphorylation. It has also been proposed that MYBBP1A is part of a set of proteins involved in the progression of mitosis, since the reduction in MYBBP1A expression caused by RNA interference (RNAi) causes both a delay in the progression of mitosis and failures in the assembly and stability of the mitotic spindle [16].

During mitosis, the nucleus breaks down, and the nucleolar proteins are translocated to the cytoplasm. Among these proteins is MYBBP1A, which, as mentioned, induces the acetylation of p53, and this interaction is essential at the G1 postmitotic control point. In this context, MYBBP1A participates in the activation of the control point machinery when mitosis is abnormally prolonged [33].

### 2.6. Role of MYBBP1A in Senescence

Several experimental data suggest that MYBBP1A could be involved in cellular senescence. First, it has been shown that MYBBP1A regulates senescence through physical interaction with two key factors in the senescence process: p53 and the RealA/p65 subunit of NF-kB [22]. Second, it has been stated that the loss of MYBBP1A affects the expression of genes involved in the cell cycle and in response to DNA damage, some of which are involved in cell senescence as well [5]. Finally, it has been described that MYBBP1A is translocated to the nucleoplasm in response to DNA damage, subsequently decreasing its levels in senescent cells [34]. In addition, an inverse regulation of MYBBP1A and AKT phosphorylation (pAKT(Ser473)) characteristic of the pre-senescent state after etoposide administration was identified in vitro. This MYBBP1A^low^pAKT(Ser473)^high^ pattern was also observed in a cohort of primary oropharyngeal squamous cell carcinoma (OPSCC) patients, which was correlated with shorter progression-free or overall survival (OS). Interestingly, most of these patients were treated with first-line or adjuvant radiotherapy, and MYBBP1A silencing in FADU cells increases clonogenic survival after irradiation, which could partially contribute to the treatment failure of tumors enriched by MYBBP1A^low^pAKT^high^ pre-senescent cells [34].

We have observed (data not published) that a decrease in the levels of MYBBP1A protein reduces entry into ras/p53-induced senescence in mouse models. This decrease allows for an approximately 40% bypass of oncogene-induced senescence, which is perhaps related to the role of MYBBP1A as a transcription factor inhibitor.

### 2.7. Role of MYBBP1A in the Regulation of Intracellular Energy Status

It has been suggested that MYBBP1A is an important component of a sensor that connects the machinery of the cell cycle with the energy of the cell and is located mainly in the nucleolus [35]. To survive, cells reduce the energy consumption and stop the cell cycle when there is an energy deficiency. In response to the absence of glucose, which translates into an energy shortage, the energy-dependent, nucleolar silencing complex (eNoSC), consisting of the proteins NML, SIRT1 and SUV39H1, evaluates the intracellular energy status based on the NAD+/NADH ratio. In situations of energy shortage, which are defined by a high proportion of NAD+/NADH, eNoSC is activated through SIRT1 and suppresses the transcription of ribosomal RNA (rRNA). This leads to a reduction in the amount of nucleolar RNA, which causes the translocation of MYBBP1A to the nucleoplasm. Finally, the translocation of MYBBP1A increases the acetylation of p53. In this way, eNoSC acts as a detector and transducer of the intracellular energy state, while MYBBP1A performs the role of effector by increasing p53 acetylation to activate it.

Thus, the eNoSC-MYBBP1A path has emerged as an alternative to the LKB1-AMPK pathway. Both routes lead to the suppression of ribosome biogenesis in response to energy descent by different mechanisms; in the case of the LKB1–AMPK pathway, the suppression occurs through the inhibition of mTOR activity. In addition, AMPK also induces the activation of p53 by increasing the phosphorylation of serine residues 15 and 17 [36,37]. The biological significance of the existence of these two routes and the independent role of each is not clear, although each can compensate for the other, because each can improve the efficiency of the other.

MYBBP1A also modulates the cellular metabolism through other pathways, such as the c-MYB–PGC-1α axis. c-MYB regulates the energy status of cells by enhancing the transcription of PGC-1α, as c-MYB activation causes an increase in PGC-1α mRNA levels [38]. Likewise, the loss of MYBBP1A activates PGC-1α both directly and indirectly. Directly, it has been published that MYBBP1A binds to PGC-1α, negatively regulating its activity as a transcriptional coactivator [20]. In addition, the results of the coimmunoprecipitation assay of our cellular model suggest the same, since we observed the binding between MYBBP1A and PGC-1α under normal conditions and the absence of such interactions in cell lines with reduced levels of MYBBP1A (data not published). In this way, the reduction or loss of MYBBP1A increases the activity of PGC-1α through two different mechanisms; the PGC-1α protein is activated by MYBBP1A reduction, and PGC-1α transcription is increased by the activation of c-MYB. On the other hand, it has been reported that the phosphorylation of PGC-1α by p38 MAPK prevents the binding of MYBBP1A to PGC-1α, preventing its repression to PGC-1α [20]. The activation of PGC-1α has a tremendous impact on the global regulation of the cellular metabolism, as PGC-1α promotes the activation of genes involved in the tricarboxylic acid cycle (TCA cycle), oxidative phosphorylation, fatty acid beta-oxidation, mitochondrial biogenesis and mitochondrial reactive oxygen species [38].

In mice that are hypomorphic for Prep1, the decrease in levels of MYBBP1A is related to an increase in the expression of PGC-1α and GLUT4, both at the mRNA and protein levels [9]. Further evidence showing the activation of PGC-1α by MYBBP1A reduction is the reduction in the expression of glycolysis genes; it has been reported that PGC-1α inhibits glycolysis by decreasing the stability of HIF1α at the protein level, leading to a decrease in the expression of genes that code for enzymes or other proteins related to glycolysis and lactate production [39]. In addition to inhibiting glycolysis, the activation of PGC-1α favors oxidative metabolism [20,40,41,42]. c-MYB+ cells (expressing c-MYB protein) with reduced levels of MYBBP1A present a higher rate of proliferation in medium with low glucose or with glutamine as the sole carbon source than cells with normal levels of MYBBP1A. This increase in proliferation occurs in normoxia but not in hypoxia, due to the absence of oxygen. This is because the advantage that oxygen provides to cells that base their metabolism on aerobic respiration disappears [38]. In addition, these cells are more sensitive to rotenone, a Complex I inhibitor, exclusively in the presence of oxygen, indicating that mitochondrial respiration is their main source of ATP. c-MYB+ cells with reduced levels of MYBBP1A also produce a greater amount of mitochondrial ROS in mediums with glutamine as the sole carbon source, reflecting an increase in the activity of the tricarboxylic acid cycle [38]. Therefore, the ultimate result of the activation of PGC-1α is the metabolic change of glycolysis to OXPHOS. This metabolic reprogramming is more evident when cells are in medium with glutamine as the sole carbon source, indicating that cells with reduced levels of MYBBP1A could base their metabolism on glutaminolysis, favoring cells that can adapt to situations of glucose shortage. Thus, the loss of MYBBP1A directly causes the activation of PGC-1α when PGC-1α is released and indirectly causes its activation through the activation of c-MYB, which induces metabolic reprogramming to OXPHOS [38]. These changes increase the sensitivity to inhibitors of the respiratory chain, suggesting a therapeutic alternative to this type of tumor [38].

In addition to regulating the biogenesis of mitochondria and their functions, such as mitochondrial respiration, PGC-1α regulates gluconeogenesis. By binding to the transcription factor FOXO1, PGC-1α activates the transcription of gluconeogenic genes, including G6PC and PCK1 [43,44].

By regulating these metabolic processes in tumor cells, PGC-1α increases the survival of tumor cells, increasing also the probability of metastasis in restrictive microenvironments [45]. However, the relevance of the role of PGC-1α in tumor processes lies not only in its function as a metabolic sensor, but also in the regulation of its activity by oncogenes and transcription factors that are involved in tumor processes and in the acquisition of metabolic plasticity. For example, the transcription factor MITF induces the expression of PGC-1α by increasing the mitochondrial capacity of a subgroup of melanomas [46]. In contrast, the oncogene MYC represses the expression of PGC-1α in pancreatic tumor cells favoring glycolytic metabolism [47]. MYC plays a central role in the metabolic plasticity of tumor cells. Depending on the conditions of the tumor microenvironment, MYC promotes aerobic glycolysis by activating the expression of glycolytic enzymes and by inhibiting the expression of PGC-1α, or induces oxidative metabolism by activating the expression of genes involved in the mitochondrial oxidation of glutamine [47,48,49,50]. In the context of metabolic plasticity, c-MYB could act in a manner contrary to MYC by activating the expression of PGC-1α under glucose-limiting conditions, which would lead to an increase in mitochondrial respiration and gluconeogenesis. In this case, the initiation of the stem cells’ properties, elicited by the activation of a MYB-dependent transcription, will be independent of PGC1a [51].

The reduction in MYBBP1A also induces the activation of mTOR (data not published), a kinase that acts as a signaling node, integrating the signals of the microenvironment. In this way, mTOR participates in the regulation of the survival, proliferation and metabolism of tumor cells, based on the availability of amino acids and oxygen, and the energy status of the cells [52,53]. It has been described that both the PI3K signaling pathway and the Ras-MAPK pathway activate mTOR, whereas the AMPK pathway inhibits its action [53].

### 2.8. Role of MYBBP1A in the Synthesis of Ribosomes

The function of MYBBP1A has also been studied in the context of the expression of ribosomal genes [54,55]. MYBBP1A is associated with the RNA polymerase I complex, and it acts as a negative regulator of rDNA transcription and as an active part of the ribosome biogenesis machinery [54]. Gain and loss of function studies suggest that MYBBP1A plays a double role in rRNA metabolism. First, it regulates the start of transcription, and second, it participates in the proper processing of pre-rRNA. The overexpression of MYBBP1A reduces the burden of RNA polymerase I on ribosomal genes. In contrast, a reduction in MYBBP1A results in the accumulation of the rRNA precursor 47S pre-RNA in vivo. It has been proposed that the accumulation of this precursor is a consequence of increases in transcription, which results from a lack of repressed expression of ribosomal genes, and failures in rRNA processing. Furthermore, it has been proposed that MYBBP1A regulates the expression of ribosomal genes by performing an epigenetic function [56,57]. Specifically, it has been proposed that MYBBP1A maintains the rDNA repeats silenced by its association with HDAC1/2, since the reduction in MYBBP1A decreases the levels of DNA methylation and histone markers associated with gene silencing; these effects alter the occupation of the promoters of some genes, such as UBF and HDACs, and increase the expression of rRNA. Finally, considering that the transcription of the ribosomal genes, the processing of the pre-rRNA and the assembly of the ribosomes are cellular processes that consume a high amount of energy, the rate of ribosome biogenesis is directly related to cell proliferation [54]. MYBBP1A is mainly associated with pre-ribosomal complexes in proliferating cells, where it ensures the correct processing of rRNA and the assembly of necessary factors for the biogenesis of ribosomes. Both low levels of ribosome synthesis in response to stress signals and a reduced ribosome demand lead to the dismantling of pre-ribosomal particles and the translocation of MYBBP1A to the nucleoplasm. Although a small fraction of MYBBP1A remains in the nucleolus, where it represses the activity of RNA polymerase I, most units of MYBBP1A are located in the nucleoplasm (Figure 1B).

### 2.9. MYBBP1A and Cancer

The gene encoding MYBBP1A is located on the short arm of chromosome 17, specifically in region *17p13.2*. Originally, MYBBP1A was reported as mapping to *17p13.3*, where the loss of heterozygosity is observed with high frequency (50%–80%) in different types of cancer, including sporadic breast and ovarian cancer, medulloblastomas, astrocytomas, osteosarcomas, leukemias, bladder cancer and lung cancer [58]. This loss of heterozygosity suggests the presence of one or more tumor suppressor genes in this region. Currently, although MYBBP1A overexpression has been associated with poor prognosis in hepatocellular carcinoma [59], it has been found to act as a tumor suppressor gene in several contexts. For example, the reduction in MYBBP1A expression in the NIH3T3 cell line induces increased cell growth and increases RasV12-mediated tumorigenesis [5,37]. A double function of MYBBP1A has also been identified in the proliferation and migration of head and neck squamous cell carcinoma cells [60]. A significant reduction in Mybbp1a levels has been observed in recurrent tumors of mice in comparison to the levels in primary tumors, with the same result observed in 50% of analyzed human tumor samples [60]. Surprisingly, the silencing of MYBBP1A in the SCC7 mouse cell line and in several human cell lines of this type of carcinoma increased cell migration but decreased cell growth [5,37]. When studying the effect of reducing the expression of MYBBP1A in vivo in xenograft models, we observed an increase in tumor size and the appearance of metastases only when injecting cells expressing c-MYB [51]. This result again supports the theory that a reduction in MYBBP1A increases the population of tumor stem cells through the activation of c-MYB [51], as it has been described that tumor stem cells are responsible for the processes of relapse and metastasis [61,62,63,64]. For this reason, it has been proposed that tumor cells with reduced levels of MYBBP1A could represent a subpopulation of slow-growing but highly migratory cells involved in relapse and metastasis.

In vivo and in vitro experiments performed on breast cancer cell lines with a normal expression of p53 showed an increase in tumorigenesis, colony formation and resistance to anoikis when the expression of MYBBP1A was silenced. Thus, MYBBP1A may have an important role in the prevention of tumor formation in relation to the activation of p53.

Tissue microarray assays in primary tumors of oropharyngeal cancer patients show a significant correlation between low levels of MYBBP1A expression and high levels of p-AKT Ser473 in patients with poor disease-free survival and poorer overall survival [34,59]. Although more studies are needed, the authors of this study suggest that the observed correlations could be indicators of a high risk of current treatment failure in patients with oropharyngeal cancer.

It has also been described that MYBBP1A is a negative regulator of sirtuin 7 (SIRT7) [65]. It has been observed that MYBBP1A binds to SIRT7 both in vitro and in vivo and inhibits the deacetylase activity of SIRT7 on H3K18Ac. In this process, the N- and C-terminal domains of SIRT7 and the C-terminal domain of MYBBP1A are involved. As the deacetylase activity of SIRT7 on H3K18Ac is necessary for the maintenance of the fundamental properties of tumor cells, the inhibition of SIRT7 by MYBBP1A suggests that MYBBP1A functions as a tumor suppressor. In addition, it has recently been identified that MYBBP1A is part of the network of proteins that interact with tripeptidyl peptidase II (TPPII). TPPII is an aminopeptidase that participates in the regulation of the cell cycle, apoptosis and senescence and seems to promote the uncontrolled growth of tumor cells. This network is composed of TPPII, p53, MYBBP1A, CDK2, SIRT7, SIRT6 and CD147 [66,67,68]. It has been suggested that the interactions between these proteins participate in the regulation of tumorigenesis, neurodegeneration and aging. In a protein–protein interaction study, it was discovered that TPPII physically interacts with MYBBP1A and the cell cycle regulatory protein CDK2 [66,67,68].

By analyzing MYBBP1A mRNA levels in paired samples of normal and tumoral tissue, MYBBP1A expression levels have been found to be reduced mainly in the kidney, liver and pancreas tumors [38]. In addition, numerous mutations or deletions have been identified in the MYBBP1A gene in different types of human tumors, most of which are loss of function mutations [51]. Expression data from the TCGA database indicate that the reduction in MYBBP1A is especially relevant in renal tumors. In addition, the degradation of MYBBP1A is regulated by the tumor suppressor gene VHL [6], which is mutated, deleted or epigenetically silenced in approximately 85% of sporadic clear-cell, renal cell carcinoma (ccRCC) [8]. Based on these observations, a cohort of kidney cancer patients was analyzed, and the reduction in or loss of expression of the MYBBP1A protein was detected in 8% of the tumors, associated with the appearance of metastasis. Likewise, patients with tumors with low MYBBP1A expression showed a clear trend of worse prognosis, including both disease-free survival (DFS) and overall survival (OS) [51]. This worse prognosis has been confirmed in ccRCC patients from the TCGA database. This association of the loss of MYBBP1A with the appearance of metastasis and worse prognosis supports the idea that a reduction in MYBBP1A expression leads to an increase in the population of tumor stem cells [51].

In renal carcinoma cell lines, the overexpression of MYBBP1A suppresses cell growth in all cell lines used, independently of the molecular context. On the other hand, the reduction in MYBBP1A levels increases tumor properties only in cell lines that express c MYB. In c-MYB + lines, the reduction in MYBBP1A levels causes an increase in the number of Cancer Stem Cells (CSCs) the capacity of tumor-formation and the expression of genes related to the regulation of the pluripotency of tumor stem cells and the epithelium–mesenchyme transition [51]. Unlike c-MYB, which is located mainly in the nucleoplasm and is expressed primarily in the bone marrow, colon crypts and neurogenic niches [1,15,17], MYBBP1A is a protein that is predominantly located in the nucleolus and is expressed in all tissues [5].

MYBBP1A binding to the NRD domain of c-MYB [69] suggests that it could act by regulating the transcriptional activity of c-MYB. The reduction in MYBBP1A expression results in an increase in the transcriptional activity of c-MYB, since the cells have increased the mRNA levels of NANOG, CD34 and CXCR4 [51], genes transcriptionally regulated by c-MYB [5,37,70,71], and involved in the acquisition and maintenance of the stem cell phenotype [72,73]. c-MYB regulates the processes of self-renewal and differentiation, preventing the differentiation of cells [5,37,72,74,75,76]. On the other hand, surface markers specific to tumor stem cells have not been established in renal tumors; however, it has been proposed that CXCR4 is essential for the maintenance of renal tumor stem cells [77], and it could be a good candidate as a specific marker of tumor stem cells [78,79,80]. Taken together, the reduction in the expression of MYBBP1A induces the activation of c-MYB, which ultimately results in an increase in the tumor stem cell phenotype. Consistently, these results were not observed when reducing the expression of MYBBP1A in cell lines that do not express c-MYB.

### 2.10. VHL, the Third Player 

The binding of pVHL to MYBBP1A causes the degradation of MYBBP1A in an iron-dependent and proteasome-dependent manner [6]. It has also been observed that the restoration of the expression of pVHL in the human renal cell adenocarcinoma cell line 786-O leads to an increase in mitochondrial DNA content and proteins in the respiratory chain, thus favoring the production of mitochondrial ATP [37]. Based on these findings, it has been proposed that tumors deficient in VHL would present stabilized levels of MYBBP1A, leading to a greater repression of PGC-1α and to a reduction in mitochondrial respiration [20]. The reduction in MYBBP1A in cell lines that express c-MYB and do not express pVHL induces the activation of PGC-1α, confirming the central role of MYBBP1A in the regulation of mitochondrial respiration through PGC-1α. Surprisingly, an exclusive relationship was found between c-MYB and pVHL, since lines expressing c-MYB do not express pVHL and vice versa. It has been described that all members of the MYB family, including c-MYB, interact with pVHL [37]. B-MYB is also targeted by pVHL for degradation via the proteasome [19,81,82]. Altogether, these results suggest that the absence of pVHL is needed to achieve an appropriately regulated expression of c-MYB, which is a key factor involved in the MYBBP1A downregulation response. However, it is not known whether this apparently opposite effect is due to differential expression or the effect of different VHL isoforms [38,51]. Therefore, more studies are needed to understand the relationship between c-MYB and pVHL in depth.

**Figure 4:**
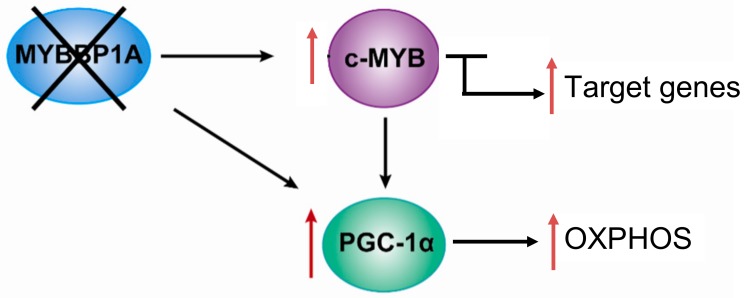
Model of the role of MYBBP1A as a tumor suppressor and its possible mechanism of action. Scheme showing the relationship between MYBBP1A, c-MYB and PGC-1α, and the impact of MYBBP1A loss on the processes regulated by these proteins. MYBBP1A is a repressor of the transcription factor c-MYB and of the co-activator of transcription PGC-1α, whose expression is regulated positively by c-MYB. The loss of MYBBP1A in cells lacking pVHL induces the activation of c-MYB and PGC-1α, causing the increase in the stem cell phenotype and the metabolic shift to OXPHOS.

Based on the previously mentioned findings, we propose the following model, in which MYBBP1A is a tumor suppressor that inhibits the activity of c-MYB and PGC-1α by binding these two transcription regulators. c-MYB is a transcription factor that regulates the expression of genes involved in the stem cell phenotype but also regulates the transcription of genes involved in other biological processes, such as the *PGC-1α* gene. *PGC-1α* is a central regulator of metabolic processes which positively regulates mitochondrial respiration and negatively regulates glycolysis. In renal tumors in which both MYBBP1A and pVHL are expressed, the expression of c-MYB is very low or null due to its mutually exclusive relationship with pVHL. The degradation of MYBBP1A would be regulated by pVHL, and MYBBP1A would bind to PGC-1α and c-MYB, repressing its activity (Figure 4). It has been proposed that the expression of pVHL is lost in approximately 85% of sporadic ccRCC [51]; therefore, this loss would produce the stabilization of c-MYB in the vast majority of renal tumors. 

In this context, 85% of ccRCC occurs when the loss of MYBBP1A causes an increase in the population of tumor stem cells and the metabolic reprogramming of glycolysis to OXPHOS. The loss of MYBBP1A expression causes the activation of c-MYB by derepression, thus increasing the expression of genes transcriptionally regulated by c-MYB that regulate the stem cell phenotype, such as *NANOG*, *CD34* and *CXCR4* [38,51]. The increase in the expression of these genes causes an increase in the population of tumor stem cells. On the other hand, the loss of MYBBP1A also directly induces the activation of PGC-1α, since PGC-1α inhibition does not occur when MYBBP1A is not available to bind it; the activation occurs indirectly because the expression of PGC-1α is activated by the action of c-MYB. Finally, the activation of PGC-1α causes metabolic changes to OXPHOS by inhibiting glycolysis and stimulating aerobic respiration, facilitating the adaptation of tumor cells to restrictive environments with a glucose shortage [38,51].

Tumor evolution will favor cells able to grow under low oxygen or low nutrient conditions. Therefore, genetic or epigenetic alterations allowing tumor cells to grow or resist under these conditions will offer selective advantages over other tumor cells selecting the clonal composition of tumors. These selective advantages will mark the landscape of final clinical observations. We found that one of these characteristics was the downregulation of MYBBP1A, allowing tumors to better survive and proliferate under low energy conditions [42]. The direct and indirect activation, through MYB, of PGC-1α, due to MYBBP1A downregulation, switches the metabolism to OXPHOS, which generates more energy under low glucose conditions. This switch may confer advantages over other tumor cells, indicating the biological relevance of the MYBBP1A downregulation in cancer.

Finally, RNA expression data of renal tumors in public databases support the proposed model. The expression of target genes of c-MYB correlates negatively with MYBBP1A expression and positively with PGC-1α expression. Interestingly, 8% of the renal tumors present low levels of MYBBP1A expression and high levels of PGC-1α and the target genes of c-MYB. These data show the existence of a percentage of tumors with reduced levels of MYBBP1A, a high transcriptional activity of c-MYB and high levels of PGC-1α expression. Likewise, these target genes are mainly related to the metabolic pathways, confirming the role of MYBBP1A in the regulation of the tumor cell metabolism through c-MYB and PGC-1α. In addition, 9% of renal tumors in the EXPO database have low expression levels of MYBBP1A and high expression levels of genes associated with the tricarboxylic acid cycle, indicating that the loss of MYBBP1A expression is associated with the induction of OXPHOS [38]. MYBBP1A downregulation in vitro also increases the cytotoxic response to oxidative channel inhibition, which may suggest a possible therapy for RCC patients [42]. The treatment with rotenone, a mitochondria respiratory channel inhibitor, rendered the cells with MYBBP1A more sensitive to cell death in an oxygen rich atmosphere, but not in hypoxia, suggesting that these cells have some degree of dependency on oxidative metabolism. This suggests that, among the different subtypes of renal cell carcinoma, MYBBP1A loss is more frequent in chromophobe renal cell carcinoma [42], which could be a suitable subgroup when targeting the oxidative pathways as an alternative therapeutic approach.

## 3. Conclusions and Perspectives

In summary, MYBBP1A is a transcription factor regulator involved in different cellular processes that can have an impact on the physiology of cancer cells, such as epigenetic regulation, mitosis, senesce, ribosomal biosynthesis and metabolic regulation. To date, MYBBP1A has been mostly found to act as a tumor suppressor gene in different molecular contexts. Our work shows that MYBBP1A acts as a tumor suppressor repressing the activity of c-MYB and PGC-1α. A decrease in MYBBP1A expression causes the activation of c-MYB, which leads to an increase in the population of tumor stem cells and is associated with the metastatic processes. In addition, the reduction in MYBBP1A levels activates PGC-1α directly by reducing the inhibition caused by the binding of MYBBP1A to PGC-1α and indirectly by increasing the transcription of PGC-1α through c-MYB. Ultimately, the activation of PGC-1α reprograms the metabolism of glycolysis to OXPHOS, facilitating the adaptation of tumor cells to microenvironments with glucose limitation. Thus, the loss of MYBBP1A provides a selective advantage to tumor cells that express c-MYB and do not express pVHL. Finally, we observed that the loss of MYBBP1A occurs in 8%–9% of renal tumors, which could be studied as possible targets of therapies using inhibitors of mitochondrial respiration.

Although a high number of MYBBP1A binding partners have been identified and our understanding of how the molecular mechanisms through MYBBP1A affects diverse cellular pathways has grown in recent years, there are still some knowledge gaps to unravel. The generation of a conditional KO mouse model could provide novel insights into the critical role of MYBBP1A in tumor development or into the impact of MYBBP1A on tumor microenvironment and its response to inhibitors of mitochondrial respiration. Likewise, studies designed to identify the kinases that target the MYBBP1A C-terminal, which contains the NLS sequence, could be crucial to completely understanding MYBBP1A regulation and/or discovering mutants with clinical relevance.

## References

[1] Tavner F.J., Simpson R., Tashiro S., Favier D., Jenkins N.A., Gilbert D.J., Copeland N.G., Macmillan E.M., Lutwyche J., Keough R.A. (1998). Molecular cloning reveals that the p160 Myb-binding protein is a novel, predominantly nucleolar protein which may play a role in transactivation by Myb. Mol. Cell Biol..

[2] Kumazawa T., Nishimura K., Kuroda T., Ono W., Yamaguchi C., Katagiri N., Tsuchiya M., Masumoto H., Nakajima Y., Murayama A. (2011). Novel nucleolar pathway connecting intracellular energy status with p53 activation. J. Biol. Chem..

[3] Yamauchi T., Keough R.A., Gonda T.J., Ishii S. (2008). Ribosomal stress induces processing of Mybbp1a and its translocation from the nucleolus to the nucleoplasm. Genes Cells.

[4] Shimizu K., Kawasaki Y., Hiraga S., Tawaramoto M., Nakashima N., Sugino A. (2002). The fifth essential DNA polymerase phi in Saccharomyces cerevisiae is localized to the nucleolus and plays an important role in synthesis of rRNA. Proc. Natl. Acad. Sci. USA.

[5] Mori S., Bernardi R., Laurent A., Resnati M., Crippa A., Gabrieli A., Keough R., Gonda T.J., Blasi F. (2012). Myb-binding protein 1A (MYBBP1A) is essential for early embryonic development, controls cell cycle and mitosis, and acts as a tumor suppressor. PLoS ONE.

[6] Lai Y., Qiao M., Song M., Weintraub S.T., Shiio Y. (2011). Quantitative proteomics identifies the Myb-binding protein p160 as a novel target of the von Hippel-Lindau tumor suppressor. PLoS ONE.

[7] Nielsen S.M., Rhodes L., Blanco I., Chung W.K., Eng C., Maher E.R., Richard S., Giles R.H. (2016). Von hippel-lindau disease: Genetics and role of genetic counseling in a multiple neoplasia syndrome. J. Clin. Oncol..

[8] Shenoy N., Pagliaro L. (2016). Sequential pathogenesis of metastatic VHL mutant clear cell renal cell carcinoma: Putting it together with a translational perspective. Ann. Oncol..

[9] Oriente F., Fernandez Diaz L.C., Miele C., Iovino S., Mori S., Diaz V.M., Troncone G., Cassese A., Formisano P., Blasi F. (2008). Prep1 deficiency induces protection from diabetes and increased insulin sensitivity through a p160-mediated mechanism. Mol. Cell Biol..

[10] Kanzleiter T., Rath M., Penkov D., Puchkov D., Schulz N., Blasi F., Schurmann A. (2014). Pknox1/Prep1 regulates mitochondrial oxidative phosphorylation components in skeletal muscle. Mol. Cell Biol..

[11] Olsen J.V., Blagoev B., Gnad F., Macek B., Kumar C., Mortensen P., Mann M. (2006). Global, in vivo, and site-specific phosphorylation dynamics in signaling networks. Cell.

[12] Nousiainen M., Sillje H.H., Sauer G., Nigg E.A., Korner R. (2006). Phosphoproteome analysis of the human mitotic spindle. Proc. Natl. Acad. Sci. USA.

[13] Cantin G.T., Yi W., Lu B., Park S.K., Xu T., Lee J.D., Yates J.R. (2008). Combining protein-based IMAC, peptide-based IMAC, and MudPIT for efficient phosphoproteomic analysis. J. Proteome Res..

[14] Imami K., Sugiyama N., Kyono Y., Tomita M., Ishihama Y. (2008). Automated phosphoproteome analysis for cultured cancer cells by two-dimensional nanoLC-MS using a calcined titania/C18 biphasic column. Anal. Sci..

[15] Keough R.A., Macmillan E.M., Lutwyche J.K., Gardner J.M., Tavner F.J., Jans D.A., Henderson B.R., Gonda T.J. (2003). Myb-binding protein 1a is a nucleocytoplasmic shuttling protein that utilizes CRM1-dependent and independent nuclear export pathways. Exp. Cell Res..

[16] Perrera C., Colombo R., Valsasina B., Carpinelli P., Troiani S., Modugno M., Gianellini L., Cappella P., Isacchi A., Moll J. (2010). Identification of Myb-binding protein 1A (MYBBP1A) as a novel substrate for aurora B kinase. J. Biol. Chem..

[17] Ramsay R.G., Gonda T.J. (2008). MYB function in normal and cancer cells. Nat. Rev. Cancer.

[18] Zhou Y.E., O’Rourke J.P., Edwards J.S., Ness S.A. (2011). Single molecule analysis of c-myb alternative splicing reveals novel classifiers for precursor B-ALL. PLoS ONE.

[19] Zhou Y., Ness S.A. (2011). Myb proteins: Angels and demons in normal and transformed cells. Front. Biosci. (Landmark Edition).

[20] Fan M., Rhee J., St-Pierre J., Handschin C., Puigserver P., Lin J., Jaeger S., Erdjument-Bromage H., Tempst P., Spiegelman B.M. (2004). Suppression of mitochondrial respiration through recruitment of p160 myb binding protein to PGC-1alpha: Modulation by p38 MAPK. Genes Dev..

[21] Diaz V.M., Mori S., Longobardi E., Menendez G., Ferrai C., Keough R.A., Bachi A., Blasi F. (2007). p160 Myb-binding protein interacts with Prep1 and inhibits its transcriptional activity. Mol. Cell Biol..

[22] Owen H.R., Elser M., Cheung E., Gersbach M., Kraus W.L., Hottiger M.O. (2007). MYBBP1a is a novel repressor of NF-kappaB. J. Mol. Biol..

[23] Hara Y., Onishi Y., Oishi K., Miyazaki K., Fukamizu A., Ishida N. (2009). Molecular characterization of Mybbp1a as a co-repressor on the Period2 promoter. Nucleic Acids Res..

[24] Jones L.C., Okino S.T., Gonda T.J., Whitlock J.P. (2002). Myb-binding protein 1a augments AhR-dependent gene expression. J. Biol. Chem..

[25] Ho L., Ronan J.L., Wu J., Staahl B.T., Chen L., Kuo A., Lessard J., Nesvizhskii A.I., Ranish J., Crabtree G.R. (2009). An embryonic stem cell chromatin remodeling complex, esBAF, is essential for embryonic stem cell self-renewal and pluripotency. Proc. Natl. Acad. Sci. USA.

[26] Ho L., Jothi R., Ronan J.L., Cui K., Zhao K., Crabtree G.R. (2009). An embryonic stem cell chromatin remodeling complex, esBAF, is an essential component of the core pluripotency transcriptional network. Proc. Natl. Acad. Sci. USA.

[27] Yang C.C., Liu H., Chen S.L., Wang T.H., Hsieh C.L., Huang Y., Chen S.J., Chen H.C., Yung B.Y., Chin-Ming Tan B. (2012). Epigenetic silencing of myogenic gene program by Myb-binding protein 1a suppresses myogenesis. EMBO J..

[28] Kumazawa T., Nishimura K., Katagiri N., Hashimoto S., Hayashi Y., Kimura K. (2015). Gradual reduction in rRNA transcription triggers p53 acetylation and apoptosis via MYBBP1A. Sci. Rep..

[29] Kuroda T., Murayama A., Katagiri N., Ohta Y.M., Fujita E., Masumoto H., Ema M., Takahashi S., Kimura K., Yanagisawa J. (2011). RNA content in the nucleolus alters p53 acetylation via MYBBP1A. EMBO J..

[30] Holmberg Olausson K., Nister M., Lindstrom M.S. (2012). p53 -Dependent and -Independent Nucleolar Stress Responses. Cells.

[31] Ono W., Hayashi Y., Yokoyama W., Kuroda T., Kishimoto H., Ito I., Kimura K., Akaogi K., Waku T., Yanagisawa J. (2014). The nucleolar protein Myb-binding protein 1A (MYBBP1A) enhances p53 tetramerization and acetylation in response to nucleolar disruption. J. Biol. Chem..

[32] Ono W., Akaogi K., Waku T., Kuroda T., Yokoyama W., Hayashi Y., Kimura K., Kishimoto H., Yanagisawa J. (2013). Nucleolar protein, Myb-binding protein 1A, specifically binds to nonacetylated p53 and efficiently promotes transcriptional activation. Biochem. Biophys. Res. Commun..

[33] Tsuchiya M., Katagiri N., Kuroda T., Kishimoto H., Nishimura K., Kumazawa T., Iwasaki N., Kimura K., Yanagisawa J. (2011). Critical role of the nucleolus in activation of the p53-dependent postmitotic checkpoint. Biochem. Biophys. Res. Commun..

[34] George B., Horn D., Bayo P., Zaoui K., Flechtenmacher C., Grabe N., Plinkert P., Krizhanovsky V., Hess J. (2015). Regulation and function of Myb-binding protein 1A (MYBBP1A) in cellular senescence and pathogenesis of head and neck cancer. Cancer Lett..

[35] Wolf D.A. (2014). Is reliance on mitochondrial respiration a “chink in the armor” of therapy-resistant cancer?. Cancer Cell.

[36] Sanli T., Steinberg G.R., Singh G., Tsakiridis T. (2014). AMP-activated protein kinase (AMPK) beyond metabolism: A novel genomic stress sensor participating in the DNA damage response pathway. Cancer Biol. Ther..

[37] Zhou G., Wang J., Zhao M., Xie T.X., Tanaka N., Sano D., Patel A.A., Ward A.M., Sandulache V.C., Jasser S.A. (2014). Gain-of-function mutant p53 promotes cell growth and cancer cell metabolism via inhibition of AMPK activation. Mol. Cell.

[38] Felipe-Abrio B., Verdugo-Sivianes E.M., Carnero A. (2019). c-MYB- and PGC1a-dependent metabolic switch induced by MYBBP1A loss in renal cancer. Mol. Oncol..

[39] Lim J.H., Luo C., Vazquez F., Puigserver P. (2014). Targeting mitochondrial oxidative metabolism in melanoma causes metabolic compensation through glucose and glutamine utilization. Cancer Res..

[40] Scarpulla R.C. (2012). Nucleus-encoded regulators of mitochondrial function: Integration of respiratory chain expression, nutrient sensing and metabolic stress. Biochim. Biophys. Acta.

[41] Scarpulla R.C. (2011). Metabolic control of mitochondrial biogenesis through the PGC-1 family regulatory network. Biochim. Biophys. Acta.

[42] Ventura-Clapier R., Garnier A., Veksler V. (2008). Transcriptional control of mitochondrial biogenesis: The central role of PGC-1alpha. Cardiovasc. Res..

[43] Puigserver P., Rhee J., Donovan J., Walkey C.J., Yoon J.C., Oriente F., Kitamura Y., Altomonte J., Dong H., Accili D. (2003). Insulin-regulated hepatic gluconeogenesis through FOXO1-PGC-1alpha interaction. Nature.

[44] Puigserver P., Spiegelman B.M. (2003). Peroxisome proliferator-activated receptor-gamma coactivator 1 alpha (PGC-1 alpha): Transcriptional coactivator and metabolic regulator. Endocr. Rev..

[45] Tan Z., Luo X., Xiao L., Tang M., Bode A.M., Dong Z., Cao Y. (2016). The role of PGC1alpha in cancer metabolism and its therapeutic implications. Mol. Cancer Ther..

[46] Vazquez F., Lim J.H., Chim H., Bhalla K., Girnun G., Pierce K., Clish C.B., Granter S.R., Widlund H.R., Spiegelman B.M. (2013). PGC1alpha expression defines a subset of human melanoma tumors with increased mitochondrial capacity and resistance to oxidative stress. Cancer Cell.

[47] Sancho P., Burgos-Ramos E., Tavera A., Bou Kheir T., Jagust P., Schoenhals M., Barneda D., Sellers K., Campos-Olivas R., Grana O. (2015). MYC/PGC-1alpha balance determines the metabolic phenotype and plasticity of pancreatic cancer stem cells. Cell Metab..

[48] Dang C.V., Kim J.W., Gao P., Yustein J. (2008). The interplay between MYC and HIF in cancer. Nat. Rev. Cancer.

[49] Li F., Wang Y., Zeller K.I., Potter J.J., Wonsey D.R., O’Donnell K.A., Kim J.W., Yustein J.T., Lee L.A., Dang C.V. (2005). Myc stimulates nuclearly encoded mitochondrial genes and mitochondrial biogenesis. Mol. Cell Biol..

[50] Wise D.R., DeBerardinis R.J., Mancuso A., Sayed N., Zhang X.Y., Pfeiffer H.K., Nissim I., Daikhin E., Yudkoff M., McMahon S.B. (2008). Myc regulates a transcriptional program that stimulates mitochondrial glutaminolysis and leads to glutamine addiction. Proc. Natl. Acad. Sci. USA.

[51] Felipe-Abrio B., Verdugo-Sivianes E.M., Saez C., Carnero A. (2019). Loss of MYBBP1A induces cancer stem cell activity in renal cancer. Cancers.

[52] Sonenberg N., Hinnebusch A.G. (2009). Regulation of translation initiation in eukaryotes: Mechanisms and biological targets. Cell.

[53] Kim L.C., Cook R.S., Chen J. (2017). mTORC1 and mTORC2 in cancer and the tumor microenvironment. Oncogene.

[54] Hochstatter J., Holzel M., Rohrmoser M., Schermelleh L., Leonhardt H., Keough R., Gonda T.J., Imhof A., Eick D., Langst G. (2012). Myb-binding protein 1a (Mybbp1a) regulates levels and processing of pre-ribosomal RNA. J. Biol. Chem..

[55] Tan B.C., Yang C.C., Hsieh C.L., Chou Y.H., Zhong C.Z., Yung B.Y., Liu H. (2012). Epigeneitc silencing of ribosomal RNA genes by Mybbp1a. J. Biomed. Sci..

[56] Hardie D.G. (2004). The AMP-activated protein kinase pathway--new players upstream and downstream. J. Cell Sci..

[57] Hardie D.G. (2004). AMP-activated protein kinase: The guardian of cardiac energy status. J. Clin. Investig..

[58] Keough R., Woollatt E., Crawford J., Sutherland G.R., Plummer S., Casey G., Gonda T.J. (1999). Molecular cloning and chromosomal mapping of the human homologue of MYB binding protein (P160) 1A (MYBBP1A) to 17p13.3. Genomics.

[59] Weng X., Wu J., Lv Z., Peng C., Chen J., Zhang C., He B., Tong R., Hu W., Ding C. (2019). Targeting Mybbp1a suppresses HCC progression via inhibiting IGF1/AKT pathway by CpG islands hypo-methylation dependent promotion of IGFBP5. EBioMedicine.

[60] Acuna Sanhueza G.A., Faller L., George B., Koffler J., Misetic V., Flechtenmacher C., Dyckhoff G., Plinkert P.P., Angel P., Simon C. (2012). Opposing function of MYBBP1A in proliferation and migration of head and neck squamous cell carcinoma cells. BMC Cancer.

[61] Hanahan D., Weinberg R.A. (2000). The hallmarks of cancer. Cell.

[62] Carnero A., Garcia-Mayea Y., Mir C., Lorente J., Rubio I.T., ME L.L. (2016). The cancer stem-cell signaling network and resistance to therapy. Cancer Treat Rev..

[63] Carnero A., Lleonart M. (2016). The hypoxic microenvironment: A determinant of cancer stem cell evolution. Bioessays.

[64] Akaogi K., Ono W., Hayashi Y., Kishimoto H., Yanagisawa J. (2013). MYBBP1A suppresses breast cancer tumorigenesis by enhancing the p53 dependent anoikis. BMC Cancer.

[65] Karim M.F., Yoshizawa T., Sato Y., Sawa T., Tomizawa K., Akaike T., Yamagata K. (2013). Inhibition of H3K18 deacetylation of Sirt7 by Myb-binding protein 1a (Mybbp1a). Biochem. Biophys. Res. Commun..

[66] Nahalkova J. (2016). The protein-interaction network with functional roles in tumorigenesis, neurodegeneration, and aging. Mol. Cell Biochem..

[67] Nahalkova J. (2015). Novel protein-protein interactions of TPPII, p53, and SIRT7. Mol. Cell Biochem..

[68] Nahalkova J., Tomkinson B. (2014). TPPII, MYBBP1A and CDK2 form a protein-protein interaction network. Arch. Biochem. Biophys..

[69] Favier D., Gonda T.J. (1994). Detection of proteins that bind to the leucine zipper motif of c-Myb. Oncogene.

[70] Quintana A.M., Zhou Y.E., Pena J.J., O’Rourke J.P., Ness S.A. (2011). Dramatic repositioning of c-Myb to different promoters during the cell cycle observed by combining cell sorting with chromatin immunoprecipitation. PLoS ONE.

[71] Quintana A.M., Liu F., O’Rourke J.P., Ness S.A. (2011). Identification and regulation of c-Myb target genes in MCF-7 cells. BMC Cancer.

[72] Sidney L.E., Branch M.J., Dunphy S.E., Dua H.S., Hopkinson A. (2014). Concise review: Evidence for CD34 as a common marker for diverse progenitors. Stem. Cells.

[73] Cheasley D., Pereira L., Lightowler S., Vincan E., Malaterre J., Ramsay R.G. (2011). Myb controls intestinal stem cell genes and self-renewal. Stem. Cells.

[74] Chambers I., Silva J., Colby D., Nichols J., Nijmeijer B., Robertson M., Vrana J., Jones K., Grotewold L., Smith A. (2007). Nanog safeguards pluripotency and mediates germline development. Nature.

[75] Chambers I., Colby D., Robertson M., Nichols J., Lee S., Tweedie S., Smith A. (2003). Functional expression cloning of Nanog, a pluripotency sustaining factor in embryonic stem cells. Cell.

[76] Zhang W., Sui Y., Ni J., Yang T. (2016). Insights into the Nanog gene: A propeller for stemness in primitive stem cells. Int. J. Biol. Sci..

[77] Gassenmaier M., Chen D., Buchner A., Henkel L., Schiemann M., Mack B., Schendel D.J., Zimmermann W., Pohla H. (2013). CXC chemokine receptor 4 is essential for maintenance of renal cell carcinoma-initiating cells and predicts metastasis. Stem. Cells.

[78] Cheng B., Yang G., Jiang R., Cheng Y., Yang H., Pei L., Qiu X. (2016). Cancer stem cell markers predict a poor prognosis in renal cell carcinoma: A meta-analysis. Oncotarget.

[79] Peired A.J., Sisti A., Romagnani P. (2016). Renal cancer stem cells: characterization and targeted therapies. Stem. Cells Int..

[80] Yuan Z.X., Shang Z., Gu J., He L. (2019). Renal targeting delivery systems. Future Med. Chem..

[81] Okumura F., Uematsu K., Byrne S.D., Hirano M., Joo-Okumura A., Nishikimi A., Shuin T., Fukui Y., Nakatsukasa K., Kamura T. (2016). Parallel Regulation of von Hippel-Lindau Disease by pVHL-Mediated Degradation of B-Myb and Hypoxia-Inducible Factor alpha. Mol. Cell Biol..

[82] Mello T., Simeone I., Galli A. (2019). Mito-nuclear communication in hepatocellular carcinoma metabolic rewiring. Cells.

